# Prognostic Difference of Pleural versus Distant Metastasis after Surgery for Lung Cancer

**DOI:** 10.3390/jcm10214846

**Published:** 2021-10-22

**Authors:** Kyla D. Joubert, Olugbenga T. Okusanya, Summer Mazur, John P. Ryan, Chigozirim N. Ekeke, Matthew J. Schuchert, Adam C. Soloff, Rajeev Dhupar

**Affiliations:** 1 Department of Cardiothoracic Surgery, University of Pittsburgh School of Medicine, 200 Lothrop Street, Pittsburgh, PA 15213, USA; joubertkd2@upmc.edu (K.D.J.); okusanya@gmail.com (O.T.O.); mazursn2@upmc.edu (S.M.); ryanjp3@upmc.edu (J.P.R.); ekekecn@upmc.edu (C.N.E.); schuchertmj@upmc.edu (M.J.S.); acs202@pitt.edu (A.C.S.); 2 Surgical Services Division, Veteran’s Affairs Pittsburgh Healthcare System, University Drive C, Pittsburgh, PA 15219, USA

**Keywords:** malignant pleural effusion, metastases, lung cancer surgery, lobectomy, lung cancer survival

## Abstract

Background: Pleural metastasis in lung cancer found at diagnosis has a poor prognosis, with 5–11 months’ survival. We hypothesized that prognosis might be different for patients who have had curative-intent surgery and subsequent pleural recurrence and that survival might differ based on the location of the first metastasis (distant versus pleural). This may clarify if pleural recurrence is a local event or due to systemic disease. Methods: A database of 5089 patients who underwent curative-intent surgery for lung cancer was queried, and 85 patients were found who had biopsy-proven pleural metastasis during surveillance. We examined survival based on pattern of metastasis (pleural first versus distant first/simultaneously). Results: Median survival was 34 months (range: 1–171) from the time of surgery and 13 months (range: 0–153) from the time of recurrence. The shortest median survival after recurrence was in patients with adenocarcinoma and pleural metastasis as the first site (6 months). For patients with pleural metastasis as the first site, those with adenocarcinoma had a significantly shorter post-recurrence survival when compared with squamous cell carcinoma (6 vs. 12 months; HR = 0.34) and a significantly shorter survival from the time of surgery when compared with distant metastases first/simultaneously (25 vs. 52 months; HR = 0.49). Conclusions: Patients who undergo curative-intent surgery for lung adenocarcinoma that have pleural recurrence as the first site have poor survival. This may indicate that pleural recurrence after lung surgery is not likely due to a localized event but rather indicates systemic disease; however, this would require further study.

## 1. Introduction

Lung cancer is responsible for the largest percent of cancer-related deaths in the United States, and unfortunately, it is commonly first diagnosed as stage IV disease [1]. Some patients present with a malignant pleural effusion (MPE) as the first finding of cancer, and prognosis is quite poor, with most studies describing 5–11 months of median survival [2,3]. Pleural drainage with systemic chemo/immunotherapy is the mainstay, and there are few options for localized treatment. Unlike other cancers that develop MPEs (e.g., breast cancer, lymphoma), lung cancer is thought to extend to the pleura through both local and systemic routes. Accordingly, some may consider that localized spread after surgery may have a different natural course than systemic spread. While MPE at the time of diagnosis is frequently widely metastatic and associated with short survival, what is less well understood is the prognosis for patients who develop MPE during surveillance after curative-intent surgery and if there is a difference in patients who have pleural recurrence first versus distant disease first. Understanding these differences may aid in selecting patients for novel localized or systemic treatments [4,5].

We hypothesized that patients that have undergone curative-intent surgery for lung cancer and are found on surveillance to have pleural recurrence would have a different prognosis from those found with pleural disease at diagnosis, as this may represent a localized event. Additionally, we hypothesized that there may be a difference in survival based on the first site of metastasis (pleural versus distant). This unique cohort of patients provides information that will help practitioners in determining prognosis and treatments.

## 2. Methods

### 2.1. Patients

**Figure 1:**
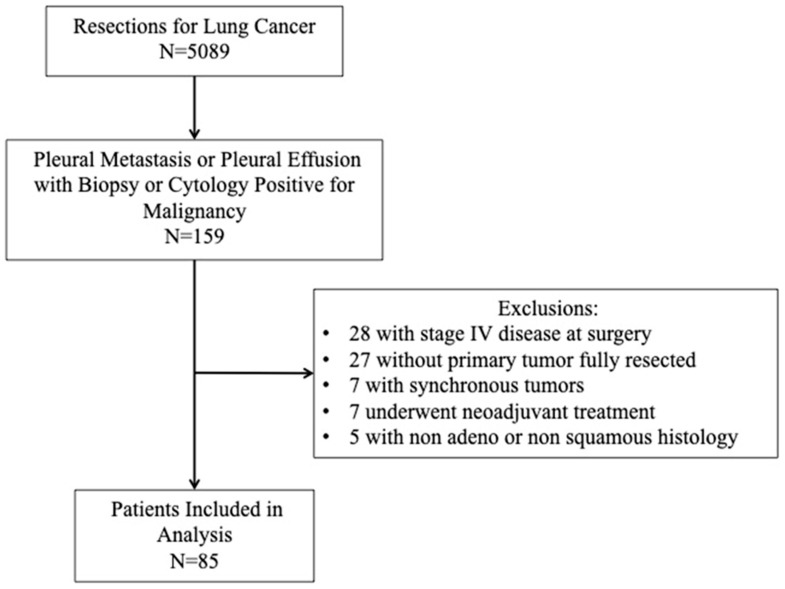
Consort diagram describing the patients included in the study.

This study was approved by the Institutional Review Board of the University of Pittsburgh. Individual patient consent was waived. We utilized the Lung Cancer Database of the University of Pittsburgh, which includes all patients at our facilities who had a lung resection from 2002 to 2020. Patients in this database were followed at 3–6-month intervals with CT scans of the chest. We selected patients who underwent curative-intent resection for non-small-cell lung cancer (NSCLC) and subsequently developed pleural metastasis. During this time, 5089 lung resections were performed, and 159 patients (3.1%) were identified as having biopsy-proven pleural metastasis or cytology-positive pleural effusion. We excluded patients who were found to be stage IV at the time of surgery, those in which the primary malignancy was not fully resected or identified, those who received neoadjuvant treatment, and non-adenocarcinoma or non-squamous histology. We also excluded patients lost to follow-up; however, we do not have an estimate of how many patients this is over this time period. Ultimately, a retrospective review of 85 patients who met the criteria was then performed (Figure 1). Clinical and pathologic staging were defined by the 8th edition of the UICC/AJCC lung cancer staging system [6]. We segregated the patients into two groups based on the following: the metastases patterns were those found on radiographic surveillance (CT or PET CT) to have isolated pleural metastasis or pleural metastasis as the first site of recurrence (Group 1), or those found on surveillance to have non-pleural metastasis first or both pleural and distant metastases simultaneously (Group 2).

### 2.2. Statistical Analysis

The patients were analyzed based on pathology (squamous versus adenocarcinoma) and pattern of metastases (described above). Normality was assessed with the Shapiro–Wilk W test. Cases were compared to controls with respect to patient demographics, operative type, staging, and histopathology. Parametric and non-parametric variables were assessed using the Student’s *t*- and Mann–Whitney U-tests. Fisher’s exact test was used for categorical variables. Logistic regression was utilized to identify factors associated with site of metastasis. Kaplan–Meier analyses with log-rank tests were performed to identify group differences in survival. Cox regression was performed to identify multivariate predictors of survival. SPSS software (version 25.0, Armonk, NY, USA) was used to perform the statistical analyses. Two-sided values of *p* < 0.05 were regarded as statistically significant.

## 3. Results

### 3.1. Patient Characteristics

Patient characteristics are displayed in Table 1. Eighty-five patients met the criteria and were included in the study, and 47 (55%) were female. The median age was 69 (range, 41–85), and 67 (79%) had a smoking history. Only two patients were non-white; thus, race was not included in subsequent analyses. Sixty-six (78%) patients were diagnosed with adenocarcinoma, and 19 (22%) were diagnosed with squamous cell carcinoma (SCC). The majority (65%) underwent lobectomy, followed by segmentectomy (26%) and pneumonectomy (5%). Clinical stage ranged from 1A1 to 3A, and pathologic stage ranged from 1A1 to 3B.

The patients were then divided into two groups: those found on surveillance to have isolated pleural metastasis or pleural metastasis as the first site of recurrence (Group 1), or those found on surveillance to have distant metastasis first or both pleural and distant metastases simultaneously (Group 2). The groups were compared for demographics, type of operation, histopathology, and staging. Univariate analysis revealed histopathologic type to be the only factor associated with pattern of metastasis, with adenocarcinoma more often presenting with distant disease (Table 1; *p* = 0.018). In multivariate logistic regression, histopathologic type was significantly associated with pattern of metastasis (Table 2; HR = 4.51, CI = 1.17–17.3, *p* = 0.028).

### 3.2. Overall Survival after Surgery

**Figure 2:**
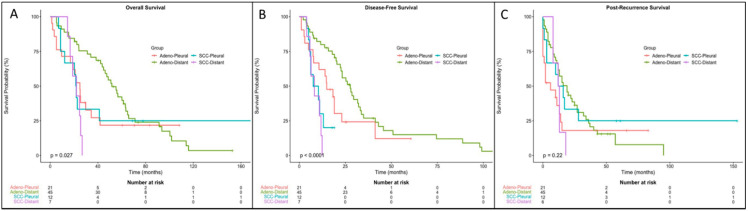
Survival for patients with adenocarcinoma versus squamous cell carcinoma. (**A**) overall survival, (**B**) disease-free survival, and (**C**) post-recurrence survival after surgery of both adenocarcinoma (Adeno) and squamous cell carcinoma (SCC) groups by log-rank test. Adeno-Pleural/SCC-Pleural: isolated pleural metastasis or pleural metastasis as the first site of recurrence (Group 1); Adeno-Distant/SCC-Distant: non-pleural metastasis first or both pleural and distant metastases simultaneously (Group 2).

**Figure 3:**
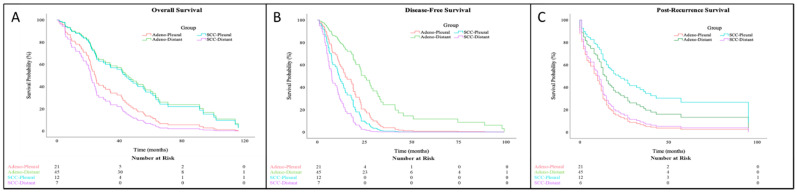
Kaplan–Meier survival curves representing (**A**) overall survival, (**B**) disease-free survival, and (**C**) post-recurrence survival from adenocarcinoma (Adeno) and squamous cell carcinoma (SCC) groups by log-rank test. Adeno-Pleural/SCC-Pleural: isolated pleural metastasis or pleural metastasis as the first site of recurrence (Group 1); Adeno-Distant/SCC-Distant: non-pleural metastasis first or both pleural and distant metastases simultaneously (Group 2).

Median overall survival was 34 months (range: 1–171) from the time of surgery. Patients with a smoking history and higher pathologic stage had worse overall survival (Table 3; *p* = 0.003 and *p* = 0.015, respectively). There was no significant difference in overall survival based on sex, histopathologic type, type of operation, and clinical stage after the curative-intent surgery. There was a significant difference in overall survival based on histology and pattern of metastasis (Figure 2A; X^2^(3) = 9.2, *p* = 0.027). Post-hoc comparison between all four groups found a significant difference in survival between distant adenocarcinoma (median survival: 51.7 months) and distant SCC (median survival: 22.0 months, *p* < 0.001). All other post-hoc comparisons between groups were non-significant. A comparison of patients with adenocarcinoma in Group 1 to those with adenocarcinoma in Group 2 showed Group 1 survived half as long from the time of surgery (24.9 versus 51.7 months, *p* = 0.076). Overall survival after surgery between these two groups was not significant in Kaplan–Meier analysis; however, when covarying for other clinical factors, Cox regression showed a significant difference in survival (Figure 3A; HR = 0.49, CI = 0.27–0.93, *p* = 0.028). Smoking history was again associated with worse survival (Table 4).

### 3.3. Disease-Free Survival after Surgery

Median disease-free survival after lung resection was 19 months (range: 1–110). Patients with a smoking history and with SCC had shorter disease-free survival (Table 3; *p* < 0.001). There was also a significant difference in disease-free survival based on pathology and pattern of metastasis (Figure 2B; *p* < 0.001), and when comparing patients with adenocarcinoma in Group 1 versus Group 2, those in Group 1 had a significantly shorter median disease-free survival (14.9 vs. 27.7 months; *p* = 0.036). Cox regression showed that patients with adenocarcinoma in Group 1 had shorter disease-free survival compared to patients in Group 2 (Figure 3B; HR = 0.43, CI = 0.23–0.82, *p* = 0.011). Smoking history remained a significant predictor of disease-free survival (Table 4).

### 3.4. Post-Recurrence Survival

Median survival after recurrence was 13 months (range: 0–153). There was shorter post-recurrence survival with increasing pathologic stage (*p* = 0.027) and history of smoking (Table 3; *p* = 0.049). Kaplan–Meier analysis found no significant difference in post-recurrence survival (Figure 2C; *p* = 0.22). However, Cox regression analysis showed that patients with adenocarcinoma in Group 1 had a significantly shorter post-recurrence survival compared to patients with SCC in Group 1 (Figure 3C; HR = 0.34, CI = 0.14–0.92, *p* = 0.034). Mirroring Kaplan–Meier analysis, smoking history and pathologic stage were factors with a significant effect on post-recurrence survival (Table 4).

## 4. Discussion

For early-stage NSCLC, surgical resection is the current standard of care for eligible patients [7,8,9] The 5-year survival after appropriate cancer resection is only 60%, mostly due to post-operative recurrence [8,10,11]. A majority of recurrences happen within two years, and mean survival after surgery may range from 1 to 34 months [12,13]. Unfortunately, 75% of all patients with NSCLC are diagnosed at an advanced or metastatic stage, and 40% of these patients will have an MPE [2,14]. Malignant pleural disease at the time of diagnosis predicts 5–11 months’ survival; however, these studies are based on a diverse collection of patients who have undergone a variety of treatments or none at all [3,15,16,17].

It is less well understood what the survival is for a patient with pleural recurrence after curative-intent surgery [2,6]. In lung cancer, pleural recurrence after surgery is thought to be either a local or systemic event, but this is often unclear. The present study revealed that in a cohort of 85 patients under surveillance after curative-intent surgery for NSCLC who had pleural recurrence that (1) median survival after surgery was 34 months and after recurrence was 13 months; (2) pattern of metastasis for patients with adenocarcinoma is associated with survival, with pleural recurrence first having a much shorter survival after surgery than patients with a distant recurrence first or synchronously (25 vs. 52 months after surgery); and (3) patients with adenocarcinoma who have pleural recurrence first have a much shorter post-recurrence survival than similar patterns of recurrence due to SCC (6 vs. 12 months). This implies that pleural recurrence is not a localized phenomenon after surgery but rather indicates systemic disease.

Treatment decisions are often guided by expected survival, which can be related to the location of the disease. In a retrospective series by Porcel et al., 556 patients with newly diagnosed lung cancer were analyzed for survival based on the location of metastases. Clinical stage ranged from I to IV, and only 16% of these patients underwent surgery. Ninety-four (17%) patients were diagnosed with an MPE. Of those with MPEs, median survival was only 5.5 months [17]. In comparison to their study, which included a heterogeneous patient population, we found that approximately 3% of patients who underwent resection for lung cancer had biopsy-proven pleural metastasis, and in our similarly sized patient cohort, the survival was much longer after pleural recurrence (median of 13 months). This indicates that the cohort of patients undergoing surgery might survive longer for a variety of reasons, probably due to the selection bias of a population who are able and willing to undergo lung resection. Importantly, it allows us to better understand the prognosis for this unique patient cohort in comparison to the general NSCLC population that more commonly have pleural disease at the time of diagnosis.

Because lung cancer originates in the pleural cavity, pleural spread can occur by local and systemic means. Other studies have examined survival based on pattern of metastases, but none have addressed survival for pleural versus distant metastasis. Hung et al. retrospectively reviewed 179 patients who underwent surgical resection for lung adenocarcinoma and had recurrence, excluding those who received neoadjuvant treatments and those with stage IV disease. With a median follow-up of 47 months, the median disease-free interval was 18 months. Among these patients, 30 (16.8%) had pleural seeding/effusion, and they found no significant difference in post-recurrence survival based on the location of metastasis [9]. Although their study focused on risk factors for recurrence after resection for adenocarcinoma and they did not specifically analyze the survival for pleural recurrence, it is one of the few studies to present data on the presence of MPE after resection and compare survival based on location. Because survival after pleural recurrence was not specifically reported in this series, we cannot draw a conclusion regarding the prognostic implications of pleural recurrence during surveillance following curative-intent lung resection.

Our cohort is unique and had a slightly better prognosis than the general NSCLC population with MPE, either due to earlier detection and treatment or a selection bias of more fit patients with better access to healthcare. However, pleural recurrence has a poor prognosis, and if it were a localized phenomenon, we might have expected a longer survival for these patients when compared with those who had distant metastases first. Instead, pleural recurrence seems to indicate systemic disease with short survival. Because these patients may be found to have systemic disease earlier, it allows us to consider if systemic treatments in conjunction with localized treatments in the pleural space for palliation should be considered as is done for bone and brain metastases [18,19]. Nonetheless, the current findings inform us about a group of patients with a poor prognosis after surgery, and this may be important for selecting patients to be treated with clinical trials or palliative pleural-based therapies.

There are several limitations of this study. The data were collected retrospectively from a single institution and are subject to selection bias. Specifically, only patients with pathologically proven pleural metastasis were included, which excludes patients with radiographic findings only. We chose to do this because a radiographic recurrence is subject to interpretation by a radiologist and is not definitive for recurrence, especially in patients who have had surgery. Additionally, we compared our cohort to data from other studies and not those seen at our institution. It is likely that patients with metastatic pleural disease were not recognized in the database or were lost to follow-up. Finally, this was a heterogeneous and very small patient population without complete data for mutational studies and systemic therapies. A power analysis (not shown) does not find the population to be adequate to answer the posed question; however, given the unique nature of this group of patients, we considered reporting these findings to still be of value.

In conclusion, patients who undergo curative-intent surgery for lung adenocarcinoma that have pleural recurrence as the first site have poor survival. Possibly this indicates poor biology systemic disease rather than a localized spread; however, this would need to be evaluated by a more in-depth study of the tumors and a larger cohort of patients. When localized therapies become available, this information may help to guide treatment decisions.

## Figures and Tables

**Table 1:** Characteristics of patients with pleural recurrence of adenocarcinoma or squamous cell carcinoma.

	Number of Patients	Group 1	Group 2	*p*-Value
Total Number of Patients	85	33 (38.8)	52 (61.2)	0.554
Age		70.0 (16)	73.5 (11)	
Gender				
Male	38 (44.7)	19 (57.6)	19 (36.5)	0.074
Female	47 (55.3)	14 (42.4)	33 (63.5)	
Smoking History				
Yes	67 (78.8)	29 (87.9)	38 (73.1)	0.172
No	18 (21.2)	4 (12.1)	14 (26.9)	
Histopathological Type				
Adenocarcinoma	66 (77.6)	21 (63.6)	45 (86.5)	0.018
Squamous Cell	19 (22.4)	12 (36.4)	7 (13.5)	
Type of Operation				
Lobectomy	55 (64.7)	22 (66.7)	33 (63.5)	0.892
Segmentectomy	22 (25.9)	8 (24.2)	14 (26.9)	
Pneumonectomy	4 (4.7)	2 (6.2)	2 (3.8)	
Other	4 (4.7)	1 (3.0)	3 (5.8)	
Clinical Stage				
1A1	4 (4.7)	3 (9.1)	1 (1.9)	0.505
1A2	22 (25.9)	7 (21.2)	15 (28.8)	
1A3	27 (31.8)	8 (24.2)	19 (36.5)	
1B	14 (16.5)	7 (21.2)	7 (13.5)	
2A	5 (5.9)	3 (9.1)	2 (3.8)	
2B	8 (9.4)	3 (9.1)	5 (5.9)	
3A	5 (5.9)	2 (6.1)	3 (5.8)	
Pathologic Stage				
1A1	3 (3.5)	1 (3.0)	2 (3.8)	0.458
1A2	10 (11.8)	5 (15.2)	5 (5.9)	
1A3	4 (4.7)	1 (3.0)	3 (5.8)	
1B	29 (34.1)	8 (24.2)	21 (40.4)	
2A	3 (3.5)	2 (6.1)	1 (1.9)	
2B	18 (21.2)	7 (21.2)	11 (21.2)	
3A	16 (18.8)	7 (21.2)	9 (17.3)	
3B	2 (2.4)	2 (6.1)	0 (0.0)	

Values are represented as n (%) and median (IQR). *p*-value is from Mann–Whitney U and Fisher’s exact tests. Group 1: isolated pleural metastasis or pleural metastasis as the first site of recurrence; Group 2: non-pleural metastasis first or both pleural and distant metastases simultaneously.

**Table 2:** Characteristics predicting pattern of metastasis via multivariate logistic regression.

	HR	95% CI	*p*-Value
Age	0.998	0.95–1.05	0.949
Sex	0.391	0.14–1.12	0.081
Smoking history	0.490	0.13–1.84	0.294
Histopathological type	4.510	1.17–17.3	0.028
Clinical Stage	1.364	0.90–2.05	0.140
Pathologic Stage	0.752	0.54–1.04	0.093
Type of operation			
Lobectomy	−	−	0.862
Segmentectomy	0.258	0.008–8.1	0.441
Pneumonectomy	0.367	0.033–4.09	0.415
Other	0.372	0.027–5.19	0.462

Hazard ratio (HR), confidence interval (CI).

**Table 3:** Analysis of survival by log-rank test.

Overall Survival				Disease-Free Survival			Post-Recurrence Survival		
	MST (Months)	95% CI	*p*-Value	MST (Months)	95% CI	*p*-Value	MST (Months)	95% CI	*p*-Value
Sex									
Male	24.0	19.3–28.6	0.640	12.4	19.1–15.6	0.110	13.0	8.7–17.3	0.675
Female	44.7	29.4–59.9		23.3	19.6–27.0		13.0	9.4–16.5	
Smoking History									
Yes	25.1	21.7–28.4	0.003	14.0	8.1–19.9	0.001	12.0	9.6–14.4	0.049
No	71.1	19.0–123		34.9	24.0–46.0		32.0	15.6–48.4	
Histopathological Type									
Adenocarcinoma	44.7	33.7–55.6	0.128	23.1	17.6–28.6	<0.001	13.0	9.6–16.4	0.965
Squamous Cell	21.8	19.3–24.2		8.2	2.8–13.6		12.0	8.9–15.1	
Metastasis Pattern									
Adeno-Pleural	24.9	18.6–31.1	0.027	14.9	8.7–21.1	<0.001	6.0	0.0–13.8	0.222
Adeno-Distant	51.7	41.1–62.2		27.7	21.5–33.9		19.0	11.5–26.5	
SCC-Pleural	21.4	18.8–23.9		7.6	1.1–14		12.0	1.8–22.2	
SCC-Distant	22.0	13.7–30.2		8.2	3.6–12.8		12.0	7.9–16.0	
Type of Operation									
Lobectomy	42.3	19.6–64.9	0.391	20.0	10.4–29.6	0.052	12.0	8.6–15.4	0.880
Segmentectomy	31.1	12.5–49.7		19.0	14.0–24.0		12.0	1.7–22.3	
Pneumonectomy	22.7	19.9–25.4		6.2	0–13.1		16.0	9.6–22.4	
Other	60.1	14.7–105		25.3	9.4–41.2		15.0	1.3–28.7	
Clinical Stage									
1A1	34.4	−	0.148	23.1	−	0.062	11.0	−	0.324
1A2	34.0	8.1–59.8		20.0	16.9–23.0		13.0	6.1–19.9	
1A3	46.1	30.6–61.5		28.0	17.9–38.0		13.0	5.0–21.0	
1B	24.6	22.5–26.6		12.4	10.2–14.6		10.0	7.6–12.4	
2A	48.7	0.0–106		23.3	0.0–51.1		17.0	12.7–21.3	
2B	14.5	0.0–42.1		6.4	0.0–12.9		8.0	0.0–20.8	
3A	22.7	21.2–24.2		6.2	1.5–10.9		18.0	13.7–22.3	
Pathologic Stage									
1A1	116.0	−	0.015	20.4	−	0.220	95.0	−	0.027
1A2	34.4	13.3–55.5		22.3	14.7–29.7		11.0	0.0–23.4	
1A3	101.8	−		88.7	−		23.0	7.0–39.0	
1B	41.2	14.8–67.5		19.0	11.1–26.9		13.0	9.1–16.9	
2A	−	−		−	−		−	−	
2B	24.6	22.5–26.6		14.9	5.9–23.8		12.0	9.9–14.0	
3A	24.0	15.9–32.0		10.9	0.0–24.3		10.0	0.0–25.5	
3B	5.2	−		2.6	−		2.0	−	

Median survival time (MST), confidence interval (CI), adenocarcinoma (Adeno), squamous cell carcinoma (SCC).

**Table 4:** Survival analysis by Cox regression.

Overall Survival				Disease-Free Survival			Post-Recurrence Survival		
	HR	95% CI	*p*-Value	HR	95% CI	*p*-Value	HR	95% CI	*p*-Value
Age	1.02	0.99–1.05	0.219	1	0.98–1.03	0.902	1.03	0.99–1.06	0.102
Sex	0.96	0.56–1.65	0.890	1.07	0.61–1.89	0.804	0.99	0.57–1.73	0.992
Smoking history	3.03	1.53–6.0	0.002	3.01	1.51–5.99	0.002	2.64	1.29–5.38	0.008
Histopathological type									
Adeno-Pleural	−	−	0.056	−	−	0.004	−	−	0.087
Adeno-Distant	0.49	0.27–0.93	0.028	0.43	0.23–0.82	0.011	0.54	0.29–1.05	0.070
SCC-Pleural	0.52	0.22–1.27	0.152	1.47	0.60–3.62	0.400	0.34	0.14–0.92	0.034
SCC-Distant	1.34	0.47–4.17	0.611	2.39	0.75–7.66	0.142	0.83	0.30–2.66	0.832
Type of Operation									
Lobectomy	−	−	0.313	−	−	0.783	−	−	0.447
Segmentectomy	1.56	0.45–5.44	0.478	1.54	0.44–5.37	0.503	1.40	0.40–4.96	0.601
Pneumonectomy	2.58	0.69–9.65	0.160	1.88	0.49–7.24	0.354	2.15	0.56–8.32	0.265
Other	1.51	0.24–9.41	0.661	1.28	0.20–8.25	0.797	1.84	0.30–11.1	0.508
Clinical stage	1	0.81–1.24	0.975	1.04	0.83–1.20	0.754	0.92	0.75–1.14	0.460
Pathologic stage	1.2	1.0–1.44	0.052	1.17	0.99–1.38	0.070	1.23	1.03–1.53	0.022

Hazard ratio (HR), confidence interval (CI), adenocarcinoma (Adeno), squamous cell carcinoma (SCC).

## Data Availability

Data is available by contacting the corresponding author (RD).
